# Chemoresistance Evolution in Ovarian Cancer Delineated by Single-Cell RNA Sequencing

**DOI:** 10.3390/ijms26146760

**Published:** 2025-07-15

**Authors:** Yuanmei Wang, Zongfu Tang, Haoyu Li, Run Zhou, Hao Wu, Xiaoping Cen, Yi Zhang, Wei Dong, Huanming Yang

**Affiliations:** 1 College of Life Sciences, University of Chinese Academy of Sciences, Beijing 100049, China; 2 Department of Gastroenterology and Hepatology, University Medical Center Groningen, University of Groningen, 9700 RB Groningen, The Netherlands; 3 HIM-BGI Omics Center, Hangzhou Institute of Medicine (HIM), Chinese Academy of Sciences, Hangzhou 310022, China; 4 Department of Biology, University of Copenhagen, 2100 Copenhagen, Denmark; 5 Institute of Genomic Medicine, Wenzhou Medical University, Wenzhou 325035, China

**Keywords:** high-grade serous ovarian cancer, single-cell RNA sequence, tumor microenvironment, transcriptome reprogramming, neoadjuvant chemotherapy

## Abstract

High-grade serous ovarian cancer (HGSOC) is an aggressive gynecological malignancy characterized by intraperitoneal spread and chemotherapy resistance. Chemotherapies have demonstrated limited effectiveness in HGSOC, underscoring the urgent need to evaluate how the tumor microenvironment (TME) was reshaped by chemotherapy in different sites of tumor foci. In this study, we performed single-cell transcriptomic analysis to explore the TME in samples obtained from various sites of tumor foci, with or without the history of Neoadjuvant chemotherapy (NACT). We discovered that chemotherapy reshaped the tumor immune microenvironment, evident through the reduction in human leukocyte antigen (HLA) diversity and the increase in *PDCD1*/*CD274* in CD8_ANXA1, *LAMP3+* dendritic cell (DC_LAMP3), and *EREG*+ monocytes (mono_EREG). Moreover, cancer.cell.2, cancer-associated *C3*+ fibroblasts (CAF_C3), and Fibrocyte_CD34, which are prone to accumulate in the metastatic site and post-NACT group, harbored poor clinical outcome, reflected in the immune exclusion and tumor progression signaling. Cell–cell communication identified a stronger interaction between cancer.cell.2 and CAF_C3, as well as Fibrocyte_CD34, in post-NACT samples, indicating that chemotherapy reshapes pre-existing cell clusters in a site-dependent manner. Our findings suggest that chemotherapy and sites of foci were critical for the transcriptional reprogramming of pre-existed cell clusters. Our study offers a single-cell phenotype data substrate from which to develop a personalized combination of chemotherapy and immunotherapy.

## 1. Introduction

Ovarian cancer (OC), a malignant tumor accounting for 5% of cancer-related deaths in females [1,2], is often diagnosed at an advanced stage and exhibits widespread intraperitoneal dissemination [3,4,5,6,7]. High-grade serous ovarian cancer (HGSOC) is commonly treated with platinum-based chemotherapies and immunotherapies. Yet, despite these therapies increasing the 10-year survival rate, their overall efficacy remains limited [8,9,10,11]. Over 75% of patients experience recurrence despite initial responsiveness to chemotherapy, resulting in HGSOC remaining a highly lethal disease with a five-year survival rate below 50% and a 10-year survival rate below 15% [12,13,14]. These challenges underscore the critical need to elucidate the complex interplay among diverse cellular components that may drive poor clinical outcome.

The intricate composition of the tumor microenvironment (TME) presents significant challenges in understanding the cellular characteristics and dynamic interactions of various cell populations in HGSOC. It is well known that chemotherapy can modify gene transcription in cancer cells, providing the possibility for single-cell RNA sequencing (scRNA-seq) to interpret the effects of chemotherapy on the TME [15,16,17,18]. For example, scRNA-seq and single-cell DNA sequencing have demonstrated that chemotherapy not only induces the selection of clones, but also leads to the reprogramming of transcriptional profiles in patients with breast cancer [19]. Similarly, studies on OC have demonstrated that chemotherapy triggers stress responses in cancer cells and promotes the development of cancer-associated fibroblasts within the inflammatory state [18]. A single-cell atlas of OC revealed that memory T-cells with high Granzyme K (*GZMK*) expression are prone to developing exhausted T-cells [20,21]. However, owing to the limited analysis in previous studies, the relationship between the tumor immune microenvironment and chemotherapy remains largely unclear in patients with HGSOC.

Understanding the heterogeneity of TME following Neoadjuvant chemotherapy (NACT) is critical in HGSOC. A study has shown that stress-related cell states persist during NACT and were associated with poorer prognosis [18]. However, previous research has primarily concentrated on ascites and other metastatic lesions to uncover the mechanisms of chemoresistance in patients with HGSOC [6,22,23]. Currently, the understanding of how NACT impacts the immune microenvironment of HGSOC is still limited, particularly concerning its effects on tumor foci across various sites. Therefore, obtaining a high-resolution cellular landscape is crucial for comprehensively characterizing the TME of HGSOC at different sites, with or without the history of NACT treatment.

In this study, we utilized scRNA-seq to analyze the cellular composition in TME before or after NACT treatment across multiple sites, including primary tumors, solid metastases, ascites, and blood. Our findings revealed associations between specific cellular compositions and how patients respond to chemotherapy, which could be valuable indicators of treatment effectiveness. In summary, our research revealed new insights into how chemotherapy altered pre-existing cell clusters through transcriptional reprogramming. This provided evidence for the combined use of chemotherapy and immunotherapy.

## 2. Results

### 2.1. Landscape of HGSOC by Multiphase scRNA-seq

**Figure 1:**
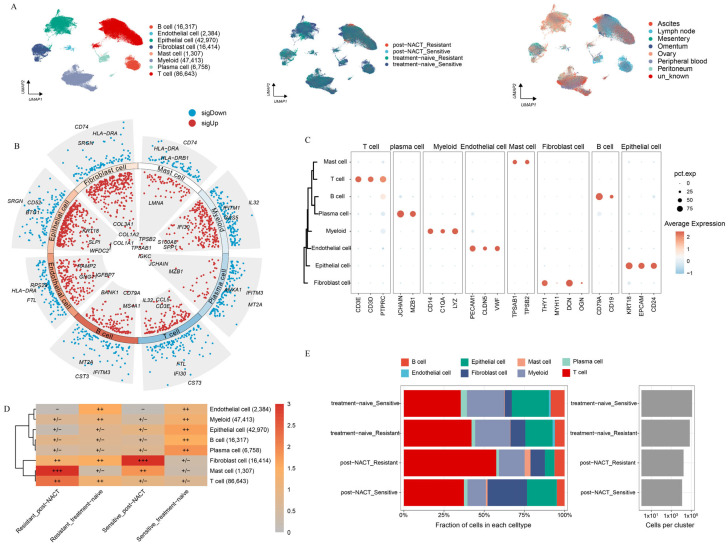
Unveiling the Single-Cell Landscape of HGSOC with or without NACT treatment. (**A**) Uniform manifold approximation and projection (UMAP) plot of HGSOC samples, with colors indicating cell type, treatment phase, and metastatic location. (**B**) Dot plots show differential gene expression across cell types, with blue for low expression and red for high expression. The three most upregulated and three most downregulated genes for each cell type are labeled. (**C**) Marker gene expression levels for each cell type are represented on a scale from blue to red. (**D**) Estimated treatment phase preferences of each cell type in HGSOC using the Ro/e metric. Ro/e index (+++, Ro/e > 3; ++, 1 < Ro/e ≤ 3; +/−, 0 < Ro/e < 0.2; and −, Ro/e = 0) to define the celltype preference in a specific treatment phase. (**E**) Proportion of each cell type distribution across treatment phases.

To delve into the intricate cellular composition of HGSOC, both with or without NACT treatment, across sites of tumor foci, we conducted scRNA-seq analysis on a diverse population of unsorted cells obtained from patients. Samples were gathered from datasets that were categorized as either sensitive or resistant to chemotherapy, regardless of whether they had undergone NACT treatment, and these samples were sourced from various metastatic sites (Supplementary Table S3). We annotated a total of 220,206 high-quality single cells into eight distinct cell clusters based on marker gene expression (Figure 1A). To confirm the accuracy of these cluster annotations, we identified the top three and bottom three different expressed genes (DEGs) for each cluster (Figure 1B). The expression patterns of marker genes across the eight clusters are shown in Figure 1C. To assess the variability in cellular composition within each sample, we examined the distribution of cell populations across all samples. In line with previous studies [24], our analysis revealed that HGSOC is a very heterogeneous disease (Supplementary Figure S1A,B).

Next, we examined the relative enrichment of the eight identified cell clusters in different tissues (Supplementary Figure S1C,D). Ascites, a common feature in HGSOC patients, contains a large amount of immune cells and is closely linked to the response to chemotherapy [1,25]. Aligned with previous research, we found that myeloid cells were mainly enriched in ascites (Supplementary Figure S1C,D). Additionally, we observed that epithelial cells were prone to enrich in primary lesions, while T-cells tended to accumulate in metastatic sites. These results highlight the distinct TME present in primary versus metastatic lesions in HGSOC (Supplementary Figure S1C,D). We further analyzed the distribution of the eight cell clusters between platinum-sensitive and platinum-resistant patients, with or without NACT treatment (Figure 1D,E). In platinum-resistant patients, a significant enrichment of T-cells was observed, while notable accumulation of mast cells was shown in post-NACT samples. Moreover, fibroblasts were the most prevalent cell type in post-NACT samples from platinum-sensitive patients, while plasma cells were primarily found in untreated platinum-sensitive samples. Endothelial cells were enriched in untreated platinum-resistant samples. Notably, all four major cell lineages—epithelial cells, myeloid cells, stromal cells, and immunocytes—were present in both treatment-naive and post-NACT samples (Supplementary Figure S1E). The enrichment patterns of four major cells exhibited no significant differences between the treatment-naive and post-NACT groups, suggesting that the TME maintained a comparable level of heterogeneity with or without the history of NACT.

### 2.2. Heterogeneity Between Epithelial Cells in HGSOC

**Figure 2:**
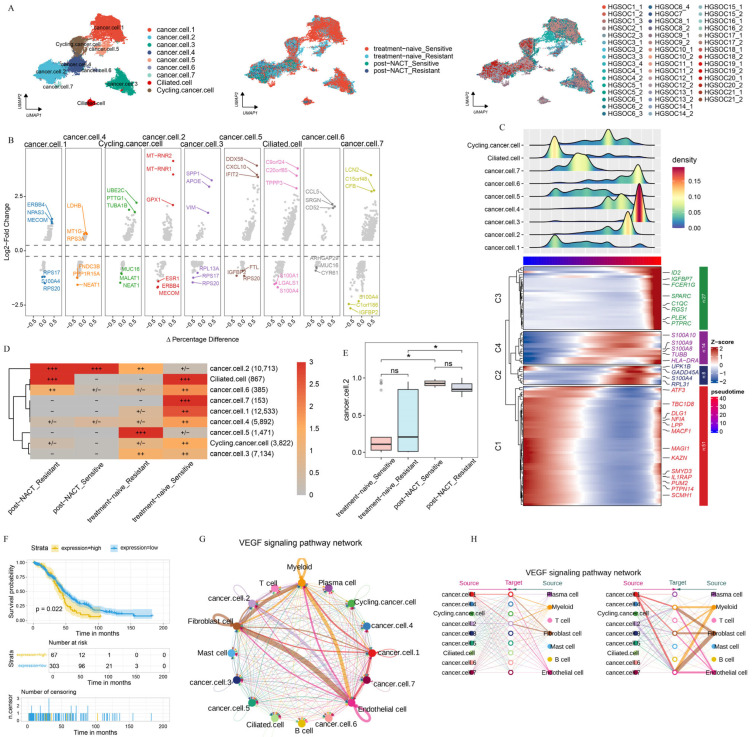
Epithelial cell heterogeneity across treatment phases. (**A**) UMAP plot showing epithelial cell subclusters, colored by cell type, treatment phase, and sample. (**B**) DEGs for each subcluster, highlighted by three downregulated and three upregulated genes. (**C**) Top: Pseudotime trajectory analysis for each subcluster. Bottom: DEGs in each module. (**D**) Treatment preference of each epithelial cluster estimated by the Ro/e metric. Ro/e index (+++, Ro/e > 3; ++, 1 < Ro/e ≤ 3; +/−, 0 < Ro/e < 0.2; and −, Ro/e = 0) to define the celltype preference in a specific treatment phase. (**E**) Box plot illustrating the percentage distribution of cancer.cell.2 cells across different treatment phases, with *p*-values determined by the Student’s *t*-test. (**F**) The overall survival ratio in patients with OV based on the expression levels of the top 10 genes, identified in cancer.cell.2 after NACT treatment. (**G**,**H**) Circle plots presenting the network strength within cancer.cell.2 (**G**) and the intricate interactions of VEGF pathways (**H**), offering insights into the cellular communication dynamics. All the data are presented as the means ± SEMs. Statistical significance is denoted by * *p* < 0.05.

Acknowledging the pivotal role of the TME in the development and progression of HGSOC, we focused on elucidating the intrinsic properties and potential functions of the epithelial cell population, considering both NACT-treated and untreated conditions across different sites. After batch-effect adjustment, we categorized the epithelial cells into three major groups (cancer.cells, ciliated.cells, and cycling.cancer.cells) and nine additional subgroups (cancer.cell.1, cancer.cell.2, cancer.cell.3, cancer.cell.4, cancer.cell.5, cancer.cell.6, cancer.cell.7, as well as ciliated.cell and cycling.cancer.cell) based on the expression level of marker genes (Figure 2A). Next, we examined the distribution of each epithelial cell subgroup across locations, with or without chemotherapy treatment (Supplementary Figure S2A,B). Furthermore, we pinpointed the distinct marker genes that are expressed by each of these epithelial cell subgroups (Figure 2B).

To delve deeper into the evolution and function of each epithelial cell subgroup, we performed the pseudotime trajectory analysis on all epithelial cell populations. The trajectory initiates on the left, where epithelial cell subgroups differentiate along the axis of tumor progression (Figure 2C). The pseudotime trajectory is split into four modules: C1 (cancer.cell.1, ciliated.cell), C2 and C4 (cancer.cell.2, cancer.cell.4, cancer.cell.5, cancer.cell.7, cycling.cancer.cell), and C3 (cancer.cell.3, cancer.cell.6). C1 was primarily marked by the expression of genes related to tumor progression and migration (*LPP* and *ATF3*) [26]. C2 and C4 were marked by the expression of genes associated with angiogenesis and tumor immune evasion (*S100A8*, *S100A9*, and *S100A10*) [27]. In C3, there was a high enrichment of genes that drive tumor cell proliferation and migration, notably C-X-C Motif Chemokine Ligand 8 (*CXCL8*) and pleckstrin (*PLEK*) (Figure 2C, Supplementary Table S4) [28]. These results suggest that epithelial cell subgroups promote tumor progression via different approaches. We also examined the enrichment patterns of these epithelial cell subgroups across various treatment phases. The results showed clear differences in cell distribution across treatment phases (Figure 2D, Supplementary Figure S2B). Cancer.cell.2 was mainly found in post-NACT samples, while cancer.cell.5 was enriched in untreated samples from chemotherapy-resistant patients. Conversely, cancer.cell.1 and cancer.cell.7 were enriched in untreated samples from chemotherapy-sensitive patients (Figure 2D), indicating that chemotherapy reshapes the ecosystems of HGSOC.

The cope number variation (CNV) analysis showed striking differences between the treatment phases, with epithelial cell subgroups having much higher CNV levels compared to T-cells (Supplementary Figure S2C,D), indicating that all epithelial cells are tumor cells. Importantly, when we counted the proportions of epithelial cell subgroups in treatment-naive and post-NACT samples, we found that only the number of cancer.cell.2 cells increased significantly after NACT treatment (Figure 2E, Supplementary Figure S2E). This finding highlights the specific response of cancer.cell.2 to NACT treatment. Furthermore, cancer.cell.2 was mainly found in metastatic lesions (Supplementary Figure S2F). In order to uncover the role of NACT in reshaping the pre-existing cell cluster, we detected the transcriptional profile of cancer.cell.2 before NACT treatment. Our analysis showed that the gene expression profile in cancer.cell.2 was linked to poorer clinical outcomes in patients after NACT treatment (Figure 2F). To thoroughly explore the intricate interactions between diverse epithelial cell subclusters and the cellular components within the TME, we conducted a cell–cell communication analysis (Figure 2G,H). Notably, among the pathways identified, the vascular endothelial growth factor (VEGF) signaling pathway stood out, given its close association with angiogenesis and immunosuppression, highlighting its significance in our study [29]. Within the VEGF signaling pathway, cancer.cell.2 showed strong communication with endothelial cells and fibroblasts (Figure 2G,H), indicating that cancer.cell.2 has a strong ability to promote angiogenesis and tumor immune escape.

A previous study about the phenotypic evolution of tumor cells revealed two distinct classes of clonal dynamics: extinction and persistence in response to NACT [19]. In line with the previous study, we found that cancer.cell.2 was presented in patients both before and after NACT, while some epithelial subgroups were extinct in response to NACT, such as cycling.cancer.cell and cancer.cell.4 in HGSOC18 (Supplementary Figure S2G). The CNV analysis revealed that the subclonal architecture remained strikingly similar between the treatment-naive group and post-NACT treatment groups (Supplementary Figure S2H). These findings lend further support to the fact that cell clusters were pre-existing and adaptively selected by the NACT treatment, while the gene expression atlas was obtained through transcriptional reprogramming.

### 2.3. T-Cell Subsets Show Treatment-Specific Patterns

**Figure 3:**
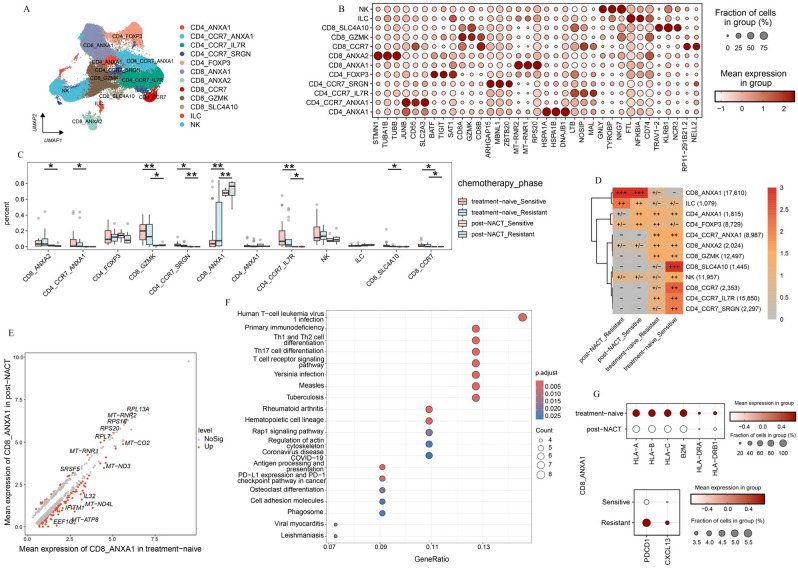
T-cell atlas of different phases. (**A**) The UMAP plot shows how the T-cell subclusters are distributed. (**B**) Dot plot presents the expression levels of DEGs across the various clusters. (**C**) Box plot illustrates the percentage of each T-cell subcluster found in different treatment phases. (**D**) The treatment phase preference of each T-cell subcluster is estimated using the Ro/e metric. Ro/e index (+++, Ro/e > 3; ++, 1 < Ro/e ≤ 3; +/−, 0 < Ro/e < 0.2; and −, Ro/e = 0) to define the celltype preference in a specific treatment phase. (**E**) The DEGs in CD8_ANXA1 are compared between patients who received NACT and those who did not. *p*-value < 0.05, avg_log2FC > 0.5. (**F**) KEGG enrichment analysis of the downregulated pathways enriched with CD8_ANXA1 after NACT. (**G**) Dot plot showing the expression patterns of selected genes across the indicated treatment phases. All the data are presented as the means ± SEMs. Statistical significance is denoted by * *p* < 0.05, ** *p* < 0.01.

The limited effectiveness of immunotherapy in HGSOC highlights the importance of examining the tumor immune microenvironment in both patients who respond to the platinum-based treatment and those who do not [9,10]. We initially examined the function of T-cell populations in HGSOC. Using unsupervised clustering, we discovered five *CD4+* subpopulations, five *CD8+* T subpopulations, one NK cell population, and one Innate Lymphoid Cell (ILC) population (see Figure 3A and Supplementary Figure S3A). We identified specific marker genes for each of these sub cell types (Figure 3B).

We observed significant differences in the abundance of T-cell subpopulations across various treatment stages (Figure 3C,D, and Supplementary Figure S3B). This highlights the unique compositions of the immune microenvironment in platinum-sensitive and platinum-resistant patients, both in the treatment-naive group and post-NACT group. Most notably, a large group of T-cells were prone to accumulate in treatment-naive samples and exhibited a pronounced decline after NACT, such as CD8_ANXA2, CD4_CCR7_ANXA1, CD8_GZMK, CD4_CCR7_IL7R, and CD8_CCR7. In contrast, we found that CD8_ANXA1 was prone to be observed in samples taken after NACT from both platinum-sensitive and platinum-resistant patients (Figure 3C,D). These results underscore the significant influence of NACT on the phenotypic changes in T-cell subpopulations in HGSOC.

We focused on CD8_ANXA1 to explore its functional shifts between treatment-naive and post-NACT groups. The DEGs analysis showed that CD8_ANXA1 had increased levels of ribosome-related genes in the post-NACT group (Figure 3E). Furthermore, the pathway enrichment analysis indicated a significant decrease in antigen processing and presentation pathways, as well as T-cell receptor signaling pathways (Figure 3F,G), pointing to functional impairments in CD8_ANXA1 after NACT. Further examination of CD8_ANXA1 showed a decrease in genes related to T-cell dysfunction (*PDCD1* and *CXCL13*) in the chemosensitive group (Figure 3G). These findings underscore the pivotal role of the chemotherapy in remodeling the tumor immune microenvironment and indicate that the low expression levels of *PDCD1* may be the reason that leads to favorable clinical outcomes.

We observed distinct tissue-specific enrichment patterns among T-cell subpopulations. Specifically, CD8_SLC4A10 and CD4_CCR7_IL7R were primarily enriched in ascites and peripheral blood, while CD4_CCR7_ANXA1 and CD4_CCR7_SRGN were mainly localized to the lymph nodes. In contrast, CD8_ANXA1 was more abundant in metastatic lesions (Supplementary Figure S3C). Our findings underscore the crucial role of sites in shaping the heterogeneous immune microenvironment of HGSOC. To delve deeper, we analyzed the functional traits of CD8_ANXA1 in both primary and metastatic lesions (Supplementary Figure S3D,E). Strikingly, we found that *MALT1*, which protected *CD274* mRNA from degradation [30], was markedly enriched in CD8_ANXA1 within metastatic lesions (Supplementary Figure S3D). The pathway enrichment analysis showed a significant downregulation of ribosome synthesis pathways (Supplementary Figure S3E), and immune-related pathways, such as Th17 cell differentiation, antigen processing and presentation, and T-cell receptor signaling, were notably downregulated in CD8_ANXA1 cells from metastatic lesions (Supplementary Figure S3F). Our findings suggest that sites play a crucial role in determining the immune resistance mechanisms in HGSOC.

In summary, the single-cell transcriptomic analysis identified two main factors affecting T-cell subpopulation distribution: chemotherapy and sites. Notably, we found that CD8_ANXA1 was highly enriched in post-NACT samples and metastatic lesions, suggesting that the enrichment of CD8_ANXA1 cells in post-NACT samples and metastatic lesions may reflect an adaptive immune-suppressive shift in the tumor microenvironment, potentially driven by therapy-induced stress or metastatic immune evasion mechanisms (Figure 3E–G, Supplementary Figure S3D,F). These findings highlight the critical roles of chemotherapy and sites in driving the phenotypic changes in T-cells and the heterogeneity of the immune microenvironment in HGSOC.

### 2.4. Myeloid Subsets Show Chemotherapy-Specific Patterns

**Figure 4:**
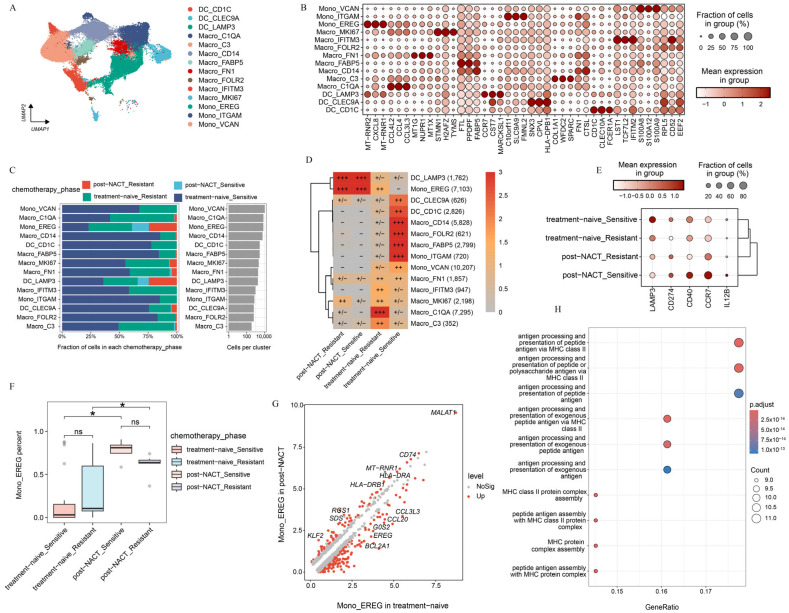
Characterization of myeloid cell phenotypes during the treatment phase. (**A**) UMAP projection of 14 myeloid cell clusters colored by cluster. (**B**) Heat map presenting the expression patterns of selected genes across the indicated clusters. (**C**) Treatment phase distribution of each myeloid cell cluster, colored by the treatment phase. (**D**) Treatment phase preference of each myeloid subcluster estimated by the Ro/e metric. Ro/e index (+++, Ro/e > 3; ++, 1 < Ro/e ≤ 3; +/−, 0 < Ro/e < 0.2; and −, Ro/e = 0) to define the celltype preference in a specific treatment phase. (**E**) Distribution of selected genes in DC_LAMP3 across the indicated phases. (**F**) The percentage of Mono_EREG among the treatment phases. (**G**) DEGs between patients treated with or without NACT in mono_EREG. (**H**) The upregulated pathways enriched in Mono_EREG after NACT treatment based on KEGG. All the data are presented as the means ± SEMs. Statistical significance is denoted by * *p* < 0.05.

Unsupervised clustering of myeloid cells revealed 15 distinct clusters, each characterized by unique gene signatures (Figure 4A). These clusters included three dendritic cell (DC) subpopulations (DC_CLEC9A, DC_CD1C, DC_LAMP3), nine macrophage subpopulations (Macro_C1QA, Macro_MKI67, Macro_C3, Macro_IFITM3, Macro_FN1, Macro_FABP5, Macro_CD14, Macro_FOLR2), and three *CD68* negative monocyte subpopulations (Mono_EREG, Mono_ITGAM, Mono_VCAN). Doublets were excluded from further analysis (Supplementary Figure S4A). Notably, the DC_LAMP3 subpopulation exhibited high expression of immunoregulatory marker genes (*LAMP3* and *CD274*) as well as maturation-related genes (*CD40*, *CCR7*, and *IL12B*) (Figure 4B, Supplementary Figure S4B,C). Consistent with previous findings, DC_LAMP3 is linked to tumor antigen presentation and has been shown to suppress DC function within the TME [31]. DC_LAMP3 was relatively enriched in the metastatic lesions of solid tumors and was less abundant in the peripheral blood and ascites [1,32] (Supplementary Figure S4D,E). Furthermore, we found that DC_LAMP3 was notably accumulated in samples with NACT (Figure 4C,D). To delve deeper into the impact of chemotherapy on the reprogramming of dendritic cell subpopulations, we detected that the expression of immunoregulatory genes in DC_LAMP3 varied from the treatment phase. We found that *LAMP3* was highly expressed in samples free from chemotherapy, while *CD274* was highly expressed in the post-NACT group. Notably, *CD40* and *IL12B* tended to be highly expressed in chemosensitive patients following NACT treatment (Figure 4E). *CD40* was a key costimulatory molecule that enhanced interactions between T-cells and myeloid cells [33]. And *IL12B* was a cytokine that promoted T-cell development [34]. These findings suggested that chemotherapy-induced dendritic cell remodeling was involved in both cell distribution and function (Figure 4D,E).

For the monocyte-macrophage system, significant distribution preferences were observed across sites and the treatment phase. The enrichment analysis revealed distinct localization patterns for various subpopulations (Supplementary Figure S4D,E). Macro_IFITM3 and Mono_VCAN were predominantly enriched in peripheral blood, whereas Macro_CD14 and Mono_ITGAM were more highly enriched in ascites. Macro_FN1, Macro_MKI67, Macro_C1QA, and Macro_C3 were enriched primarily in solid tumors, whereas Mono_EREG was localized predominantly in the metastatic lesions of solid tumors (Supplementary Figure S4D,E), suggesting that the distribution of monocyte-macrophage subpopulations varied from sites. Further analysis of monocyte-macrophage subpopulations across the treatment phase revealed that Mono_EREG had higher enrichment scores in post-NACT samples, whereas other subpopulations were enriched primarily in treatment-naive samples (Figure 4C,D,F; Supplementary Figure S4F). Gene expression profiling of Mono_EREG between the treatment-naive and post-NACT group revealed striking differences (Figure 4G). In treatment-naive samples, Mono_EREG exhibited high expression of CCL family genes (*CCL3L3*, *CCL20*), *EREG*, and *BCL2A1*, which are associated with tumor progression [35,36,37], suggesting functional impairment (Figure 4G, Supplementary Figure S4G). After receiving NACT, Mono_EREG displayed high expression of antigen presentation-related genes such as *CD74*, *HLA-DRA*, and *HLA-DRB1* [38] (Figure 4G,H, Supplementary Figure S4G). *MALT1* was also found to be highly expressed. *MALT1* protected *CD274* mRNA from degradation and promoted the proliferation and polarization of tumor-associated macrophages to create an immunosuppressive tumor microenvironment [30]. These findings suggest that chemotherapy and metastatic locations were critical for the phenotypic divergence and immunosuppressive mechanisms in HGSOC.

In summary, we identified distinct gene expression patterns and functional roles of myeloid cell subpopulations across sites with or without NACT, underscoring their context-specific contributions to HGSOC progression and transcriptome reprogramming.

### 2.5. Chemotherapy Reshapes the Phenotype of Fibroblasts in HGSOC

**Figure 5:**
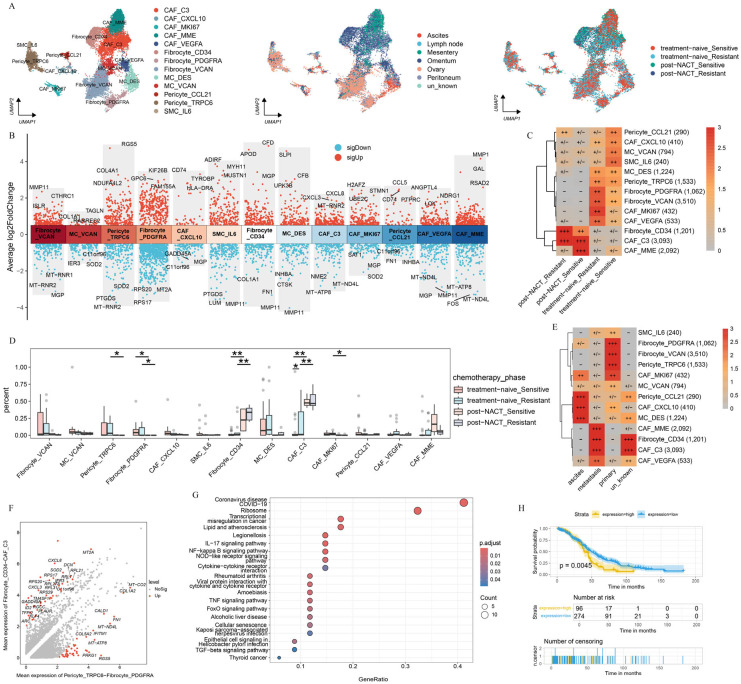
Characterization and dynamics of fibroblast subclusters in HGSOC. (**A**) UMAP plot with fibroblast subclusters colored by cell type, site, and treatment phase. (**B**) Differential expression patterns across each subcluster, labeled with three downregulated and three upregulated genes. (**C**) Treatment phase preference of each fibroblast subcluster estimated by the Ro/e metric. Ro/e index (+++, Ro/e > 3; ++, 1 < Ro/e ≤ 3; +/−, 0 < Ro/e < 0.2; and −, Ro/e = 0) to define the celltype preference in a specific treatment phase. (**D**) Box plot showing the percentage distribution of each fibroblast subcluster among treatment phases. (**E**) Site preference of each subcluster estimated by the Ro/e metric. Ro/e index (+++, Ro/e > 3; ++, 1 < Ro/e ≤ 3; +, 0.2 ≤ Ro/e ≤ 1; +/−, 0 < Ro/e < 0.2; and −, Ro/e = 0) to define the celltype preference in a specific treatment phase. (**F**) DEGs between the indicated clusters. (**G**) Kyoto Encyclopedia of Genes and Genomes (KEGG) analysis revealed that the upregulated pathways were enriched in CAF_C3 and Fibrocyte_CD34. (**H**) Kaplan–Meier analysis of patients from The Cancer Genome Atlas (TCGA) cohort with high and low gene set variation analysis (GSVA) scores on the basis of the top 26 markers of CAF_C3 and Fibrocyte_CD34. All the data are presented as the means ± SEMs. Statistical significance is denoted by * *p* < 0.05, ** *p* < 0.01.

Due to the impact of chemotherapy on gene expression [39], we further investigated the transcriptional profiles of fibroblasts from different sites before or after NACT. We divided fibroblasts into 13 cell subpopulations based on the gene expression profile (Figure 5A, Supplementary Figure S5A), including five cancer-associated fibroblast (CAF) clusters (CAF_C3, CAF_CXCL10, CAF_MKI67, CAF_MME, and CAF_VEGFA), three fibrocyte clusters (Fibrocyte_CD34, Fibrocyte_PDGFRA, and Fibrocyte_VCAN), two mesothelial cell (MC) clusters (MC_DES and MC_VCAN), two pericyte clusters (Pericyte_CCL21 and Pericyte_TRPC6), and one vascular smooth muscle cell cluster (SMC_IL6). DEGs were analyzed to infer the specific functions of each fibroblast subcluster (Figure 5B).

Next, in order to uncover the mechanism by which chemotherapy reshaped the fibroblast subcluster, we explored the distribution of fibroblast subpopulations during the treatment phase. The result presented that MC_VCAN and SMC_IL6 were predominantly enriched in platinum-sensitive samples without NACT treatment, whereas CAF_MKI67 was highly enriched in platinum-resistant samples without NACT treatment. In contrast, Fibrocyte_CD34 and CAF_C3 were preferentially enriched in post-NACT samples (Figure 5C). Further analysis revealed a significant decrease in the abundance of CAF_MKI67, Pericyte_TRPC6, and Fibrocyte_PDGFRA before NACT treatment, whereas Fibrocyte_CD34 and CAF_C3 showed the opposite trend (Figure 5D). Previous studies have reported that Fibrocyte_CD34 promotes tumor growth and enhances angiogenesis within the cancer niche during lung cancer progression [40]. In addition, we found that CAF_C3 highly expressed *CXCL3* and *CXCL8*, which are known to play critical roles in tumor progression. Specifically, *CXCL3* drives the transformation of CAFs into myofibroblastic CAFs, facilitating tumor metastasis [41], whereas *CXCL8* promotes tumor cell proliferation, EMT, and an immunosuppressive tumor microenvironment [42]. Our result presented that Fibrocyte_CD34 and CAF_C3 may be associated with the tumor progression.

We further examined the distribution of the identified fibroblast cell subpopulations across sites of tumor foci (Figure 5E). CAF_C3 and Fibrocyte_CD34 were enriched predominantly in metastatic lesions, whereas Pericyte_TRPC6 and Fibrocyte_PDGFRA were localized primarily to primary lesions. Next, we characterized the distinct features of fibroblast subclusters on the basis of the treatment phase and sites. CAF_C3 and Fibrocyte_CD34 displayed high expression levels of CXCL family genes (*CXCL3*, *CXCL8*) and *MT2A*, whereas Pericyte_TRPC6 and Fibrocyte_PDGFRA, which showed a marked decline after NACT, specifically expressed *COL1A2*, *COL5A2*, and RGS5 (Figure 5F). The pathway enrichment analysis revealed that CAF_C3 and Fibrocyte_CD34 were strongly associated with pathways related to transcriptional misregulation in cancer, NF-kappa B signaling, negative regulation of lymphocyte activation, and negative regulation of chemokine production (Figure 5G, Supplementary Figure S5D). In contrast, Pericyte_TRPC6 and Fibrocyte_PDGFRA were significantly enriched in pathways such as protein digestion and absorption, vascular smooth muscle contraction, and extracellular structure organization (Supplementary Figure S5E,F). The survival analysis revealed that the gene expression of CAF_C3 and Fibrocyte_CD34 were significantly associated with poor clinical outcomes (Figure 5H). These findings suggest that the chemotherapy and sites may reshape the function of fibroblast subclusters and affect clinical outcomes.

This analysis highlights the important role of chemotherapy and sites in the selection of pre-existing cell populations, emphasizing the critical role of the TME in guiding the development of more effective therapeutic strategies.

### 2.6. Transcriptome Reprogramming of Pre-Existing Cell Populations Following Chemotherapy

**Figure 6:**
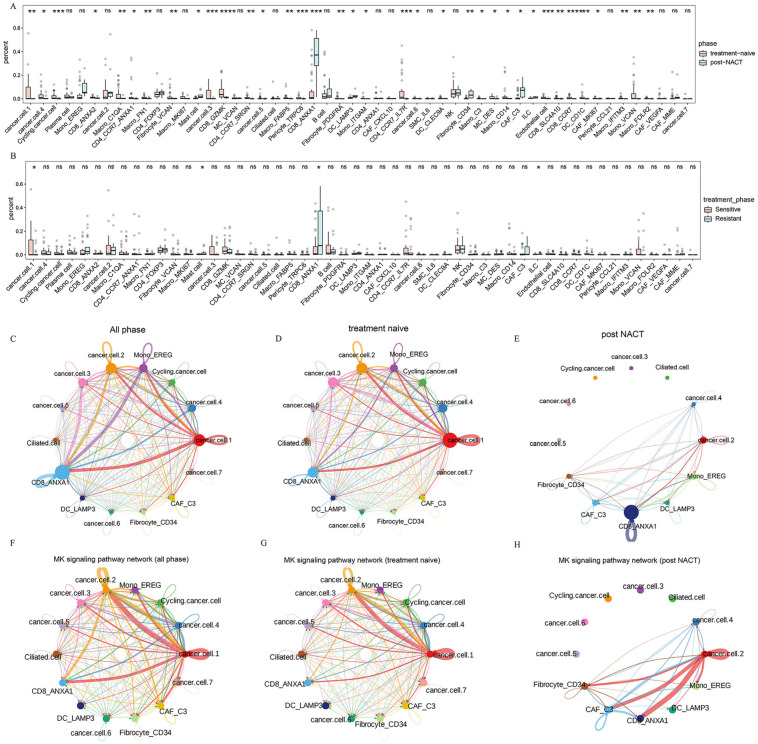
Cell–cell communication between TME cells. (**A**,**B**) The percentage distribution of each cell cluster among different treatment phases (**A**) and different treatment responses (**B**). (**C**–**E**) Circle plots demonstrating the interaction strength in all phases (**C**), treatment-naive patients (**D**), and post-NACT patients (**E**). (**F**–**H**) Circle plots demonstrating the interactions of MK signaling pathway network in all phases (**F**), treatment-naive patients (**G**), and post-NACT patients (**H**). All the data are presented as the means ± SEMs. Statistical significance is denoted by * *p* < 0.05, ** *p* < 0.01, *** *p* < 0.001, **** *p* < 0.001.

The single-cell analysis linked TME variation to chemotherapy processes that drive cell selection. We sought to identify these results with cell–cell interactions. We analyzed the distribution of all cell subsets within treatment-naive and post-NACT samples, as well as among different chemotherapy response groups (Figure 6A,B). Mono_EREG, CD8_ANXA1, DC_LAMP3, Fibrocyte_CD34, and CAF_C3 were significantly increased in the post-NACT group (Figure 6A), whereas cancer.cell.1, Mast cell, CD8_ANXA1, and ILC exhibited notable differences in their response to chemotherapy (Figure 6B). To further understand these dynamics, we performed cell–cell communication analysis to elucidate the interactions between distinct epithelial subclusters and the highly enriched cell subsets following NACT. The results revealed strong interactions between cancer.cell.2 and Mono_EREG, CD8_ANXA1 (Figure 6C, Supplementary Figure S6A). Next, we separately investigated the interactions of cell subsets within treatment-naive and post-NACT treatment samples (Figure 6D,E; Supplementary Figure S6B,C). In the treatment-naive group, epithelial subclusters, particularly cancer.cell.2, strongly interacted with Mono_EREG and CD8_ANXA1, while this interaction was reduced in the post-NACT group (Figure 6D,E; Supplementary Figure S6B,C), indicating that NACT may reshape the TME. We found that cancer.cell.2 exhibited enhanced communication with CD8_ANXA1 and fibroblasts after NACT in the MK signaling pathway network (Figure 6F–H; Supplementary Figure S6D–F). Additionally, the receptor–ligand interaction analysis revealed that in the MDK-NCL signaling network, cancer.cell.2 displayed increased communication with CD8_ANXA1, Mono_EREG, and CAF_C3 in the post-NACT group (Supplementary Figure S6D–F). The MDK-NCL signaling network inverts stromal cells into malignant phenotypes, and NCL can suppress immune activity [43]. These findings indicate the transcriptome reprogramming of pre-existing cell populations following chemotherapy.

## 3. Discussion

Although platinum-based neoadjuvant chemotherapy significantly improves survival rates in HGSOC patients, approximately 75% of patients experience relapse due to chemotherapy resistance [12,13,14]. In this study, we integrated 53 scRNA-seq datasets, including samples from platinum-resistant and platinum-sensitive patients in the treatment-naive or post-NACT phase, to systematically analyze the transcriptome reprogramming of TME dependent on chemotherapy and sites of tumor foci.

In this study, we explored the role of chemotherapy and sites in the selection of pre-existing cell populations. We revealed a significant increase in cancer.cell.2 following NACT, characterized by the high expression of S100 family genes. The S100 family is known to promote the expression of cytokines, chemokines, MMPs, and angiogenic and antiapoptotic factors, which collectively drive cell proliferation, metastasis, angiogenesis, and immune evasion [44,45]. We suggested that cancer.cell.2 was in an immune escape state. Additionally, we observed that cancer.cell.2 was predominantly distributed in metastatic lesions and strongly associated with poor prognosis in patients with OV. Our study of epithelial cells prompted us to rethink the pivotal role of chemotherapy and sites in reshaping the TME, offering new insights and references for future therapeutic strategies.

Here, we identified a distinct enrichment pattern of T-cells across the treatment phase. CD8_ANXA1 was enriched predominantly in metastatic lesions and post-NACT samples. The pathway enrichment analysis revealed significant downregulation of antigen processing and presentation pathways, as well as T-cell receptor signaling pathways, suggesting functional impairment of CD8_ANXA1. In addition, we found that HLA diversity was reduced after NACT, suggesting transcriptome reprogramming of CD8_ANXA1 following chemotherapy. Further investigation of the function of CD8_ANXA1 after NACT revealed a reduction in T-cell dysfunction-related genes (*PDCD1* and *CXCL13*) in the chemosensitive group (Figure 3G). The interaction between PD-1 and PD-L1 inhibits T-cell proliferation and cytokine secretion, negatively regulating lymphocyte activation and contributing to immune evasion [16,46], providing that chemotherapy with PD-1 inhibitor may improve the clinical outcome. It was reported that the combination of chemotherapy and immune checkpoint inhibitors has been approved in the treatment of naive metastatic non-small cell lung cancer [47]. These findings highlight the critical role of chemotherapy in reshaping the tumor immune microenvironment and emphasize the critical role of CD8_ANXA1, particularly in the context of immune checkpoint modulation.

In addition to T-cells, myeloid cells exhibited significant differences in distribution across the treatment phase. We focused particularly on DC_LAMP3 and Macro_EREG. We found that DC_LAMP3 had a higher enrichment score during the post-NACT phase. Further analysis of DC_LAMP3 suggested that they played a crucial role in promoting T-cell infiltration and differentiation in chemosensitive patients after NACT [48]. But the high expression of *CD274* inhibited the function of T-cells [49,50]. Similarly, Macro_EREG was also highly enriched in the post-NACT phase and exhibited strong antigen presentation capabilities after NACT. However, *MALT1*, which protected *CD274* mRNA from degradation, was highly expressed in Mono_EREG after NACT [30]. These results highlighted the profound impact of NACT on the transcriptome reprogramming of myeloid cells. The combination of chemotherapy and PD-L1 therapy may reverse the immunosuppressive microenvironment.

Moreover, we identified a stromal cell subpopulation that may prompt tumor progression. Our findings revealed that CAF_C3 and Fibrocyte_CD34, which were significantly enriched in the post-NACT phase, were associated with shorter overall survival.

We also explored the cell–cell communication profile among TME cells based on receptor–ligand pairs. We found that cancer.cell.2 communicated with CD8_ANXA1, Mono_EREG, CAF_C3, and Fibrocyte_CD34, which further identified that cancer.cell.2 might be an immune-escaped cell cluster. Meanwhile, the interaction was stronger in the post-NACT group compared to that in the treatment-naive group, which mainly focused on the MK signaling pathway. The MK signaling pathway was closely related to metastasis, migration, and angiogenesis [51]. As a specific type of MK signaling pathway, the role of MDK-NCL signaling network in cancer has been extensively studied. The MDK-NCL signaling network has been shown to promote the immunosuppressive environment and is associated with poor prognosis in endometrial carcinoma [43]. We suggested that the TME was in an immunosuppressive state with the history of NACT. Combining the immune checkpoint inhibitor with chemotherapy has been successful in the treatment of difficult-to-treat cancers, including lung cancer, esophagus cancer, gastric cancer, breast cancer, and cervical cancer [52]. Therefore, it is suggested that the combination of chemotherapy and immunotherapy may improve the clinical outcomes in patients with HGSOC.

In summary, our study establishes a comprehensive single-cell atlas of HGSOC, delineating how NACT induces transcriptomic reprogramming of resident cell populations to dynamically remodel the TME. These findings provide critical mechanistic insights with direct clinical implications: (1) The identified NACT-dependent TME alterations, particularly in immune cell composition and stromal interactions, offer potential biomarkers for predicting the treatment response and could guide patient stratification for chemo–immunotherapy combinations; (2) our demonstration of PD-1/PD-L1 axis activation and MDK-NCL signaling-mediated immune evasion following NACT provides a strong rationale for clinical trials testing sequential or concurrent administration of immune checkpoint inhibitors with chemotherapy; (3) the molecular signatures of therapy-resistant cell populations may enable the development of targeted adjuvant therapies tailored to individual patients’ residual disease characteristics. By elucidating these fundamental mechanisms of TME plasticity, our work not only advances the biological understanding of HGSOC progression, but also provides a translational framework to optimize therapeutic strategies in precision oncology, where TME profiling could inform real-time clinical decision-making to improve patient outcomes.

## 4. Materials and Methods

### 4.1. Public Data Sources

We obtained 53 publicly available single-cell HGSOC datasets from the Gene Expression Omnibus (GEO) database and the National Genomics Data Center. These datasets cover a wide range of samples, from treatment-naive to post-NACT, primary to metastatic lesions, and platinum-sensitive to platinum-resistant cases (Supplementary Table S1) [1,18]. Additionally, we retrieved RNA-seq datasets from the TCGA.

### 4.2. Single-Cell Data Processing

We applied additional quality control measures to ensure the retention of high-quality cells (Supplementary Table S2). Cells expressing fewer than 500 genes or with more than 12% of unique molecular identifiers (UMIs) originating from mitochondrial genes were excluded. We used Seurat (version 4.3.0.1) [53] for data preprocessing, which involved log normalization and linear regression to create the gene expression matrix. The “vst” method helped us identify the top 2000 variable features for each sample. To address batch effects across samples, we used the Harmony package (version 1.2.0) [54]. We performed dimensionality reduction using 2D UMAP through the Seurat RunUMAP function. The first 30 principal components (PCs) were used for the embedding process. For unsupervised clustering, we applied a shared nearest neighbor (SNN) algorithm based on modularity optimization, with a resolution parameter of 0.4. The resulting cell clusters were visualized using the clusterCornerAxes function from the R package scRNAtoolVis (version 0.0.7). We identified differentially expressed genes in each cell cluster using the FindAllMarkers function. The parameters were set to thresh.use = 0.5, min.pct = 0.5, and only.pos = FALSE to ensure comprehensive analysis.

### 4.3. Major Cell Type Identification

We confidently identified distinct cell types in each sample based on classical marker genes. Specifically, T-cells were defined by the expression of *CD3E, CD3D*, and *PTPRC*; plasma cells by *JCHAIN* and *MZB1*; myeloid cells by *CD14*, *C1QA*, and *LYZ*; endothelial cells by *PECAM1*, *CLDN5*, and *VWF*; mast cells by *TPSAB1* and *TPSB2*; fibroblasts by *THY1*, *MYH11*, *DCN*, and *OGN*; B-cells by *CD79A* and *CD19;* and epithelial cells by *KRT18*, *EPCAM,* and *CD24*. For each identified subcluster, we applied the Harmony package to perform batch correction, facilitating further downstream analysis.

### 4.4. Single-Cell Copy Number Variation Analysis

We used inferCNV (version 1.19.1) to analyze HGSOC scRNA-seq data, focusing on identifying large-scale somatic chromosomal CNVs. This included detecting amplifications or deletions of entire chromosomes or large chromosomal segments. The input data for inferCNV comprised expression matrix files, cell type annotation files, and gene annotation files. We designated all cells except epithelial cells as reference cells. To infer chromosomal variations, we analyzed gene expression intensities across different genomic regions in tumor cells using a threshold parameter of 0.1. For each epithelial cell subgroup, we calculated CNV scores by aggregating CNV levels within the subgroup. Finally, we generated a heatmap to visualize the regions of the tumor genome that showed amplification or deletion.

### 4.5. Pseudotime Analysis

We employed Monocle 2 (version 2.24.0) [55] to construct pseudotime trajectories for epithelial cells. This powerful tool is designed to reconstruct the temporal dynamics of cellular states by mapping their variation trajectories. To begin, we converted the Seurat object into a CellDataSet object for analysis in Monocle 2. We applied size factors to normalize the differences in mRNA levels between cells, ensuring accurate and reliable comparisons. For the analysis, we included genes that were expressed in at least 10% of the cells. To reduce dimensionality, we used UMAP. Finally, we visualized the pseudotime trajectory results using the plot_cells function.

### 4.6. Differential Expression and Gene Ontology Enrichment Analysis

To compare gene expression across cell clusters and find significantly changed genes, we used the FindAllMarkers() function in Seurat. This helped us identify marker genes that were overexpressed in each cluster. Genes with an adjusted *p*-value less than 0.05 were considered cluster-specific DEGs. We then performed enrichment analysis on the identified DEGs using clusterProfiler [56] and GSVA [57]. This analysis was complemented by functional annotation from databases such as KEGG and GO, allowing us to gain insights into the functional roles of these DEGs.

### 4.7. Survival Analysis

To find DEGs in the cell subpopulations, we used the FindAllMarkers function and picked the top-ranked genes for further survival analysis. We retrieved bulk RNA-seq data for ovarian cancer from the TCGA database and calculated subcluster feature scores using GSVA. We generated Kaplan–Meier survival curves using the survfit function from the R package Survival (version 3.5.8). Statistical significance was set at a *p*-value of less than 0.05.

### 4.8. Cell–Cell Interaction Analysis

To explore cell–cell communication mediated by ligand–receptor interactions among different cell types, we used CellChat (version 1.6.1) [58]. This tool analyzes and compares intercellular communication across various cell subpopulations by leveraging gene expression data. CellChat models these interactions by integrating ligand–receptor pairs and their associated cofactors, providing a comprehensive simulation of cell communication networks.

### 4.9. Statistics and Reproducibility

No data was excluded from our analysis. All statistical analyses were performed using R (version 4.3.2). We assessed significance using either one-sided or two-sided unpaired Student’s *t*-tests or Wilcoxon tests, with a *p*-value of less than 0.05 considered statistically significant. Although we assumed a normal distribution for the data, this assumption was not formally verified.

## 5. Conclusions

In summary, we have presented a thorough atlas detailing the landscape of HGSOC across various treatment phases, uncovering the transcriptome reprogramming of pre-existing cell populations that contribute to the reshaping of the TME in a chemotherapy-dependent manner. Furthermore, our findings shed light on the synergistic potential of combining chemotherapy with immunotherapy, offering a promising avenue to prevent tumor immune escape and effectively inhibit tumor progression. This research holds significant promise for HGSOC treatments and opens up new avenues for further exploration in the field.

### Clinical Perspectives

- A large amount of patients with HGSOC experience recurrence despite initial responsiveness to chemotherapy, suggesting that chemotherapy may reshape the tumor microenvironment.
- Chemotherapy makes a contribution to transcriptome reprogramming of pre-existing cell populations associated with reshaping of the tumor microenvironment.
- The combination of chemotherapy and immunotherapy, such as *PD-1*/*PD-L1* therapy or MDK-NCL signaling suppression therapy, may effectively inhibit tumor progression and hold significant promise for HGSOC treatments.

## Data Availability

scRNA-seq data have been deposited in the GEO database and the National Genomics Data Center [1,18].

## Supplementary Materials

The supporting information can be downloaded at: https://www.mdpi.com/article/10.3390/ijms26146760/s1.

## Abbreviations

HGSOC	high-grade serous ovarian cancer
TME	tumor microenvironment
NACT	Neoadjuvant chemotherapy
HLA	human leukocyte antigen
OC	Ovarian cancer
ScRNA-seq	single-cell RNA sequencing
GZMK	Granzyme K
DEG	different expressed gene
UMAP	uniform manifold approximation and projection
CXCL8	C-X-C Motif Chemokine Ligand
PLEK	pleckstrin
CNV	cope number variation
VEGF	vascular endothelial growth factor
ILC	Innate Lymphoid Cell
DC	dendritic cell
CAF	cancer-associated fibroblast
MC	mesothelial cell
SMC	vascular smooth muscle cell
KEGG	Kyoto Encyclopedia of Genes and Genomes
GSVA	gene set variation analysis
GEO	Gene Expression Omnibus
TCGA	The Cancer Genome Atlas
UMIs	unique molecular identifiers
PCs	principal components
SNN	shared nearest neighbor
Mono	monocytes
