# Supplementary material for: Chemoresistance Evolution in Ovarian Cancer Delineated by Single-Cell RNA Sequencing
Source: Int J Mol Sci. 2025 Jul 15;26(14):6760. doi: 10.3390/ijms26146760 (PMC12294846; doi:10.3390/ijms26146760)
Supplement: Supplementary file 1 [file ijms-26-06760-s001.zip › Supplementary Figure and figure legend.pdf]

International Journal of
Molecular Sciences

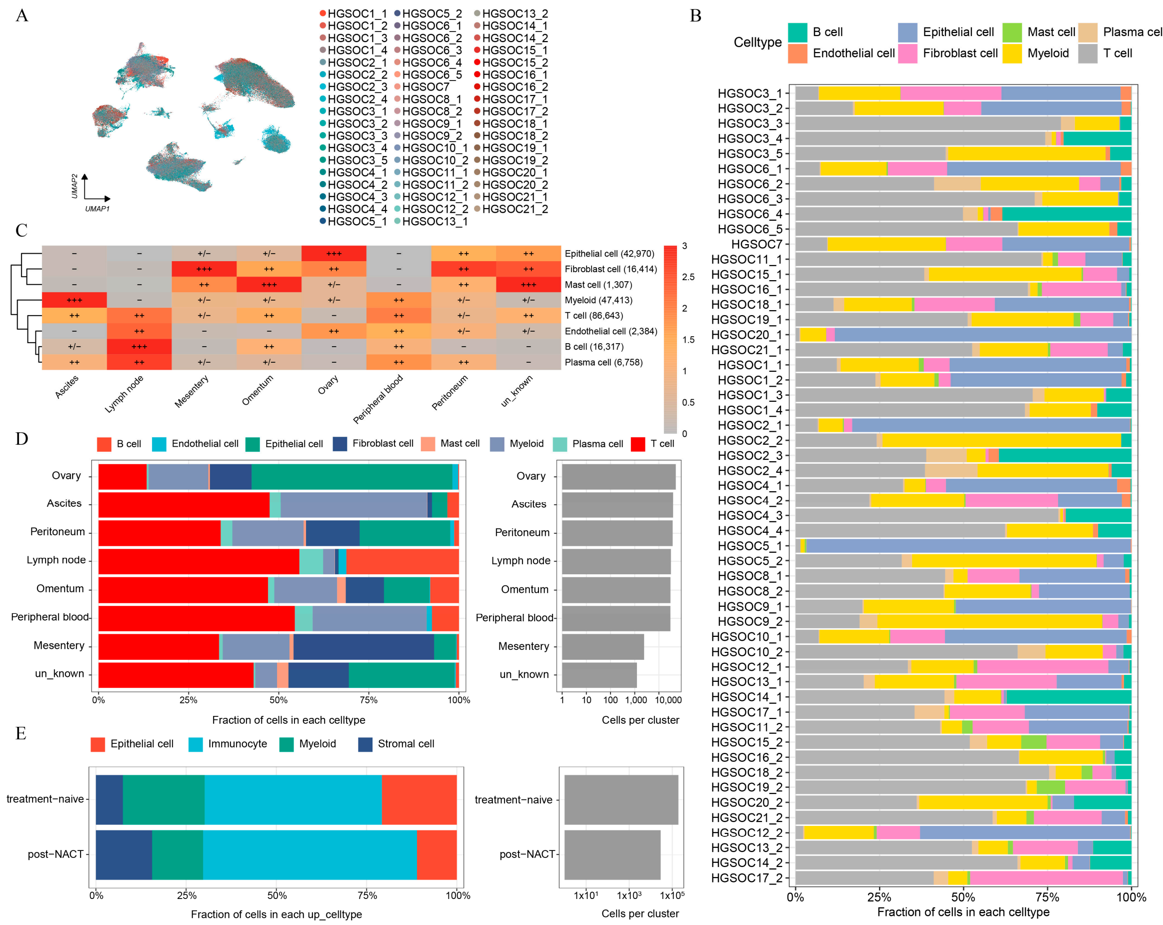

Supplementary Figure S1 Basic information of each cell type. (A). UMAP plots showing each sample distribution. (B). The cell type distribution among each sample. (C). Site preference of each stromal cluster estimated by Ro/e. D-E. Site distribution (D) and treatment phase distribution (E) of each cell type detected in HGSOC patients.

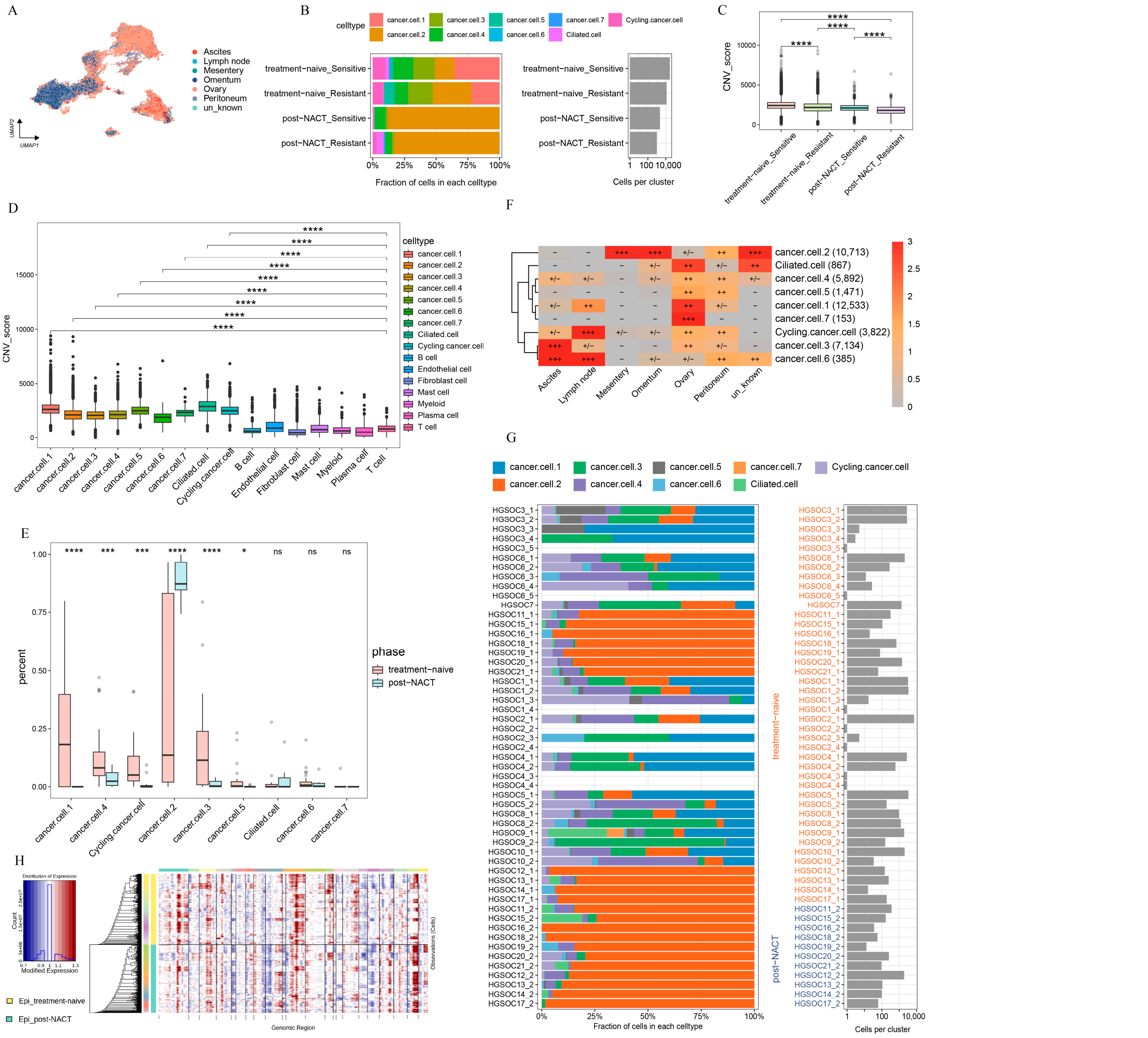

Supplementary Figure S2 Heterogeneity of epithelial cells. (A).UMAP plots showing the site distribution in epithelial cells. (B). Proportion of the epithelial subgroups in the treatment phase. C-D. CNV distribution in the treatment phase (C) and the indicated cell clusters (D). (E). The percentage distribution of epithelial cell clusters between the indicated treatment phases. (F). Site preference of each epithelial cluster estimated by the Ro/e. (G). Proportion of epithelial cell clusters in each sample. H. Heatmap showing the CNV score in different phases; CNVs in red indicate amplifications, and those in blue indicate deletions.

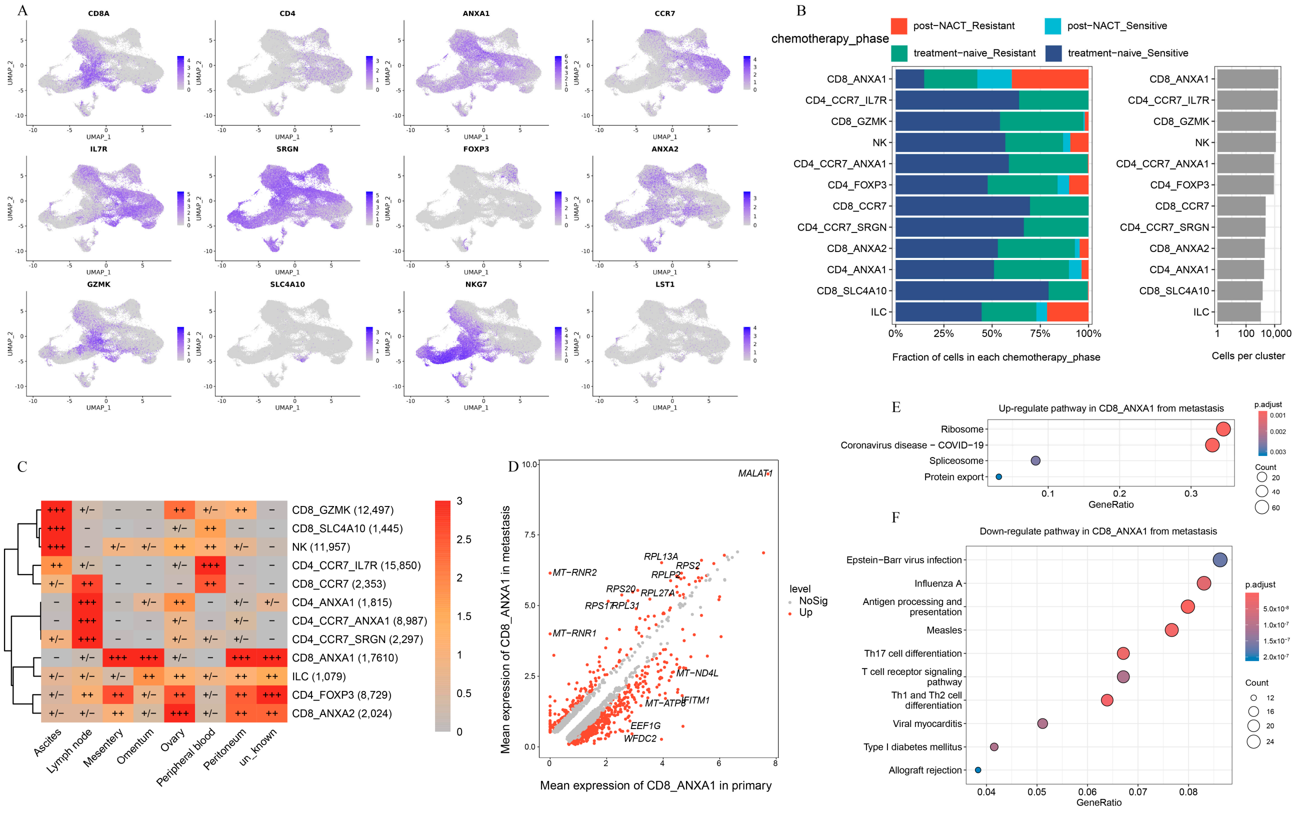

Supplementary Figure S3. (A). Clustering and characterization of T cells in HGSOC UMAP plot showing the distribution of marker genes in T cells. (B). The percentage distribution of the T subcluster in the treatment phase. (C). Site preference of each T cluster estimated by Ro/e. (D). DEGs in CD8_ANXA1 between primary and metastatic locations. (E-F). The upregulated pathways (E) and downregulated pathways (F) enriched in CD8_ANXA1 at the site of metastasis based on KEGG analysis.

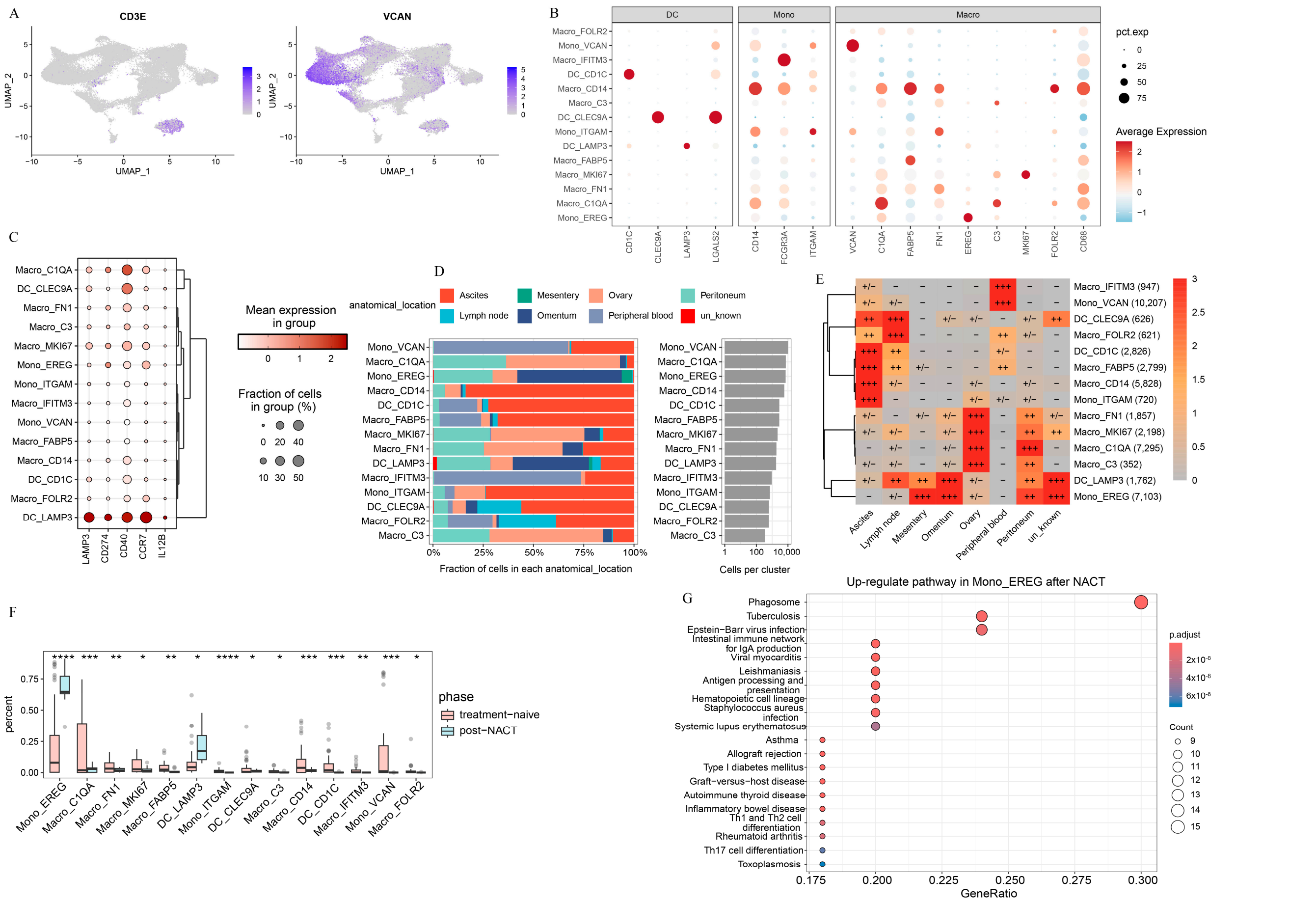

Supplementary Figure S4 Clustering and characterization of myeloid cells (A).UMAP plot showing the distribution of the indicated genes in myeloid cells. (B). Dot plot showing the marker gene expression level in each myeloid cell cluster. (C). Dot plot presenting the indicated genes expressed in each myeloid cell cluster. (D). Percent distribution of myeloid cell clusters according to site. (E). Site preference of each myeloid cluster estimated by Ro/e. (F). The percentage distribution of myeloid subclusters in the indicated groups. (G). The upregulated pathways enriched in Mono_EREG after NACT.

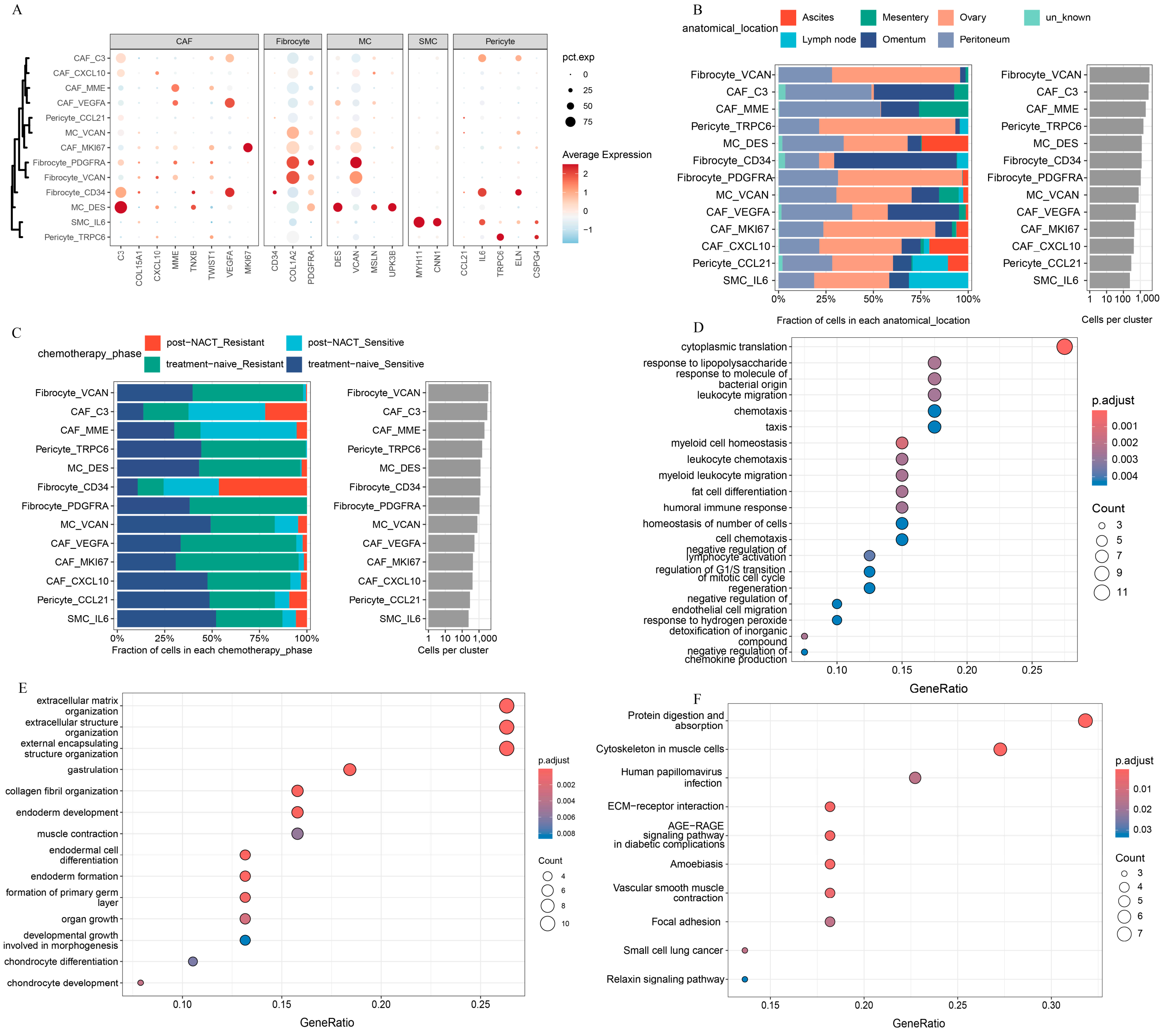

Supplementary Figure S5 Features of stromal cells. (A). Dot plot indicating the gene expression level in stromal subgroubs. (B-C). The percentage distributions of stromal cell clusters according to site (B) and treatment phase (C). (D). GO analysis revealed that the upregulated pathways enriched in CAF_C3 and Fibrocyte_CD34. (E-F). The upregulated pathways enriched in Pericyte_TRPC6 and Fibrocyte_PDGFRA were identified via GO analysis (E) and KEGG analysis (F).

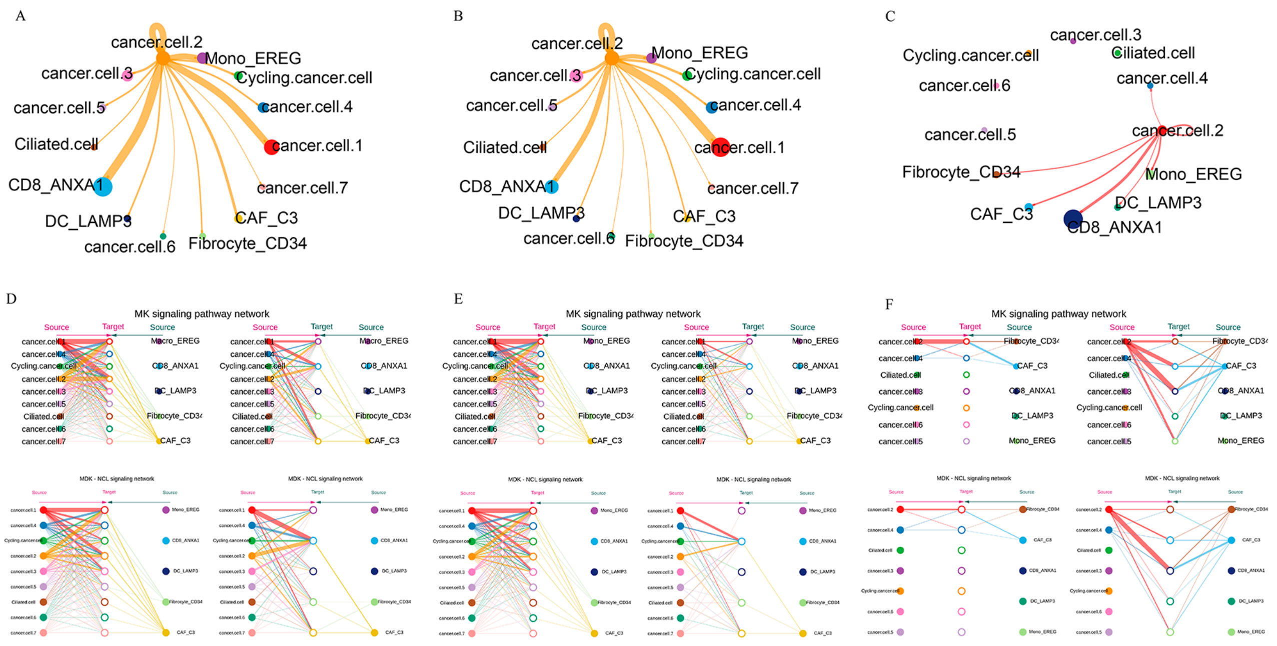

Supplementary Figure S6. Cell cell communication feature in TME. (A-C). Circle plots demonstrating the interaction strength between the indicated cell clusters in all phases (A), treatment-naïve patients (B) and post-NACT patients (C). (D–F). Weighed gene coexpression network analysis among the indicated cell subgroups in all phases (D), treatment-naïve patients (E) and post-NACT patients (F).
